# Ab Initio Molecular Dynamics Simulation Study on the Stereo Reactions between Atomic Oxygen Anion and Methane

**DOI:** 10.3390/molecules23102495

**Published:** 2018-09-29

**Authors:** Weihua Wang, Wenling Feng, Wenliang Wang, Ping Li

**Affiliations:** Key Laboratory of Life-Organic Analysis, School of Chemistry and Chemical Engineering, Qufu Normal University, Qufu 273165, China; wwh78@qfnu.edu.cn (W.W.); wlwangqf@126.com (W.W.)

**Keywords:** ion–molecule reaction, Ab initio molecular dynamics simulation, roaming reaction, stereodynamics

## Abstract

Ion–molecule reaction between atomic oxygen anion (O^−^) and methane (CH_4_) has been systematically investigated employing the on-the-fly ab initio molecular dynamics simulations. Besides the major H-abstraction process as the exothermic reaction studied widely, an endothermic pathway to produce OCH_3_^−^ and H is also observed in this study. Three typical O^−^ attack modes with reference to the pyramid structure of CH_4_ fixed in space have been considered. It was found that the internal motions of the radical products are significantly dependent on the O^−^ attack modes. As for the reaction between O^−^ and the thermally vibrating CH_4_, the major pathway to produce OH^−^ and CH_3_ is preferred by the direct H-abstraction and the minor pathway to produce H and OCH_3_^−^ is the roaming reaction via the transient negative ion [HO-CH_3_]^−^.

## 1. Introduction

As a high clean fossil energy, how to convert methane (CH_4_) to high-density energy sources and high value-added chemicals has attracted interests of researchers from the fields of the energy science and coordination chemistry [1,2,3,4,5,6,7,8]. However, as the first step of the reaction, it is difficult to activate the C-H bond of CH_4_ due to its high stability. On the other hand, as a highly reactive free radical anion, atomic oxygen anion (O^−^ at ^2^P state) can effectively activate the C-H bond, which is involved in the ion–molecule reaction. Here, ion–molecule reaction between O^−^ and molecules has a profound significance in aspects of atmospheric chemistry, combustion, and environmental pollution control [9,10,11,12,13,14,15,16,17]. As a prototype reaction for ion–molecule reaction in hydrocarbon flames, the reaction between O^−^ and methane (CH_4_) is an important example. For this reaction mechanism, Comer and Schulz reported an associative-detachment channel [18],

O^−^ + CH_4_ → CH_3_OH + e^−^ (1)

and it was proved to be an exothermic process (∆*H* = −2.43 eV [9]). In the subsequent experimental studies [19,20,21,22,23], the other reaction pathway [19,20,23,24],

O^−^ + CH_4_ → OH^−^ + CH_3_ (∆*H* = −0.26 eV) (2)

was characterized as an exothermic process, in which OH^−^ was formed via direct H-abstraction via the transition-state complex [O-H-CH_3_]^−^ [21]. On the basis of the measurements of energy and angular distributions of the product OH^−^, Carpenter and Farr proposed two mechanisms for the above reaction, namely, one is the collisions of O^−^ with H atom oriented essentially along the relative velocity vector and the other one is the collisions of O^−^ with one of the three off-axis hydrogen atoms [23]. Obviously, the reaction between O^−^ and CH_4_ possesses the stereodynamics features. However, the spatial properties of this reaction remain unclear to our best knowledge although the stereodynamics is one of the essential issues of molecular reactions. Theoretically, the kinetics of the reactions of O^−^ with CH_4_, CH_2_D_2_, and CD_4_ has been explored by using the Gaussian-1, Gaussian-2, and the complete basis set extrapolation method [21]. The minimum energy reaction path for the above reaction (2) was constructed and was characteristic of the standard double minimum pathway for ion molecule reactions. In addition, the potential surface for the relevant reaction of the O(^1^D) atom with CH_4_ has also been investigated by the ab initio Multireference single and double configuration interaction (MRDCI). The proposed product channels to yield CH_3_ + OH and CH_3_O + H can give helpful clues to the title reaction [25].

**Figure 1:**
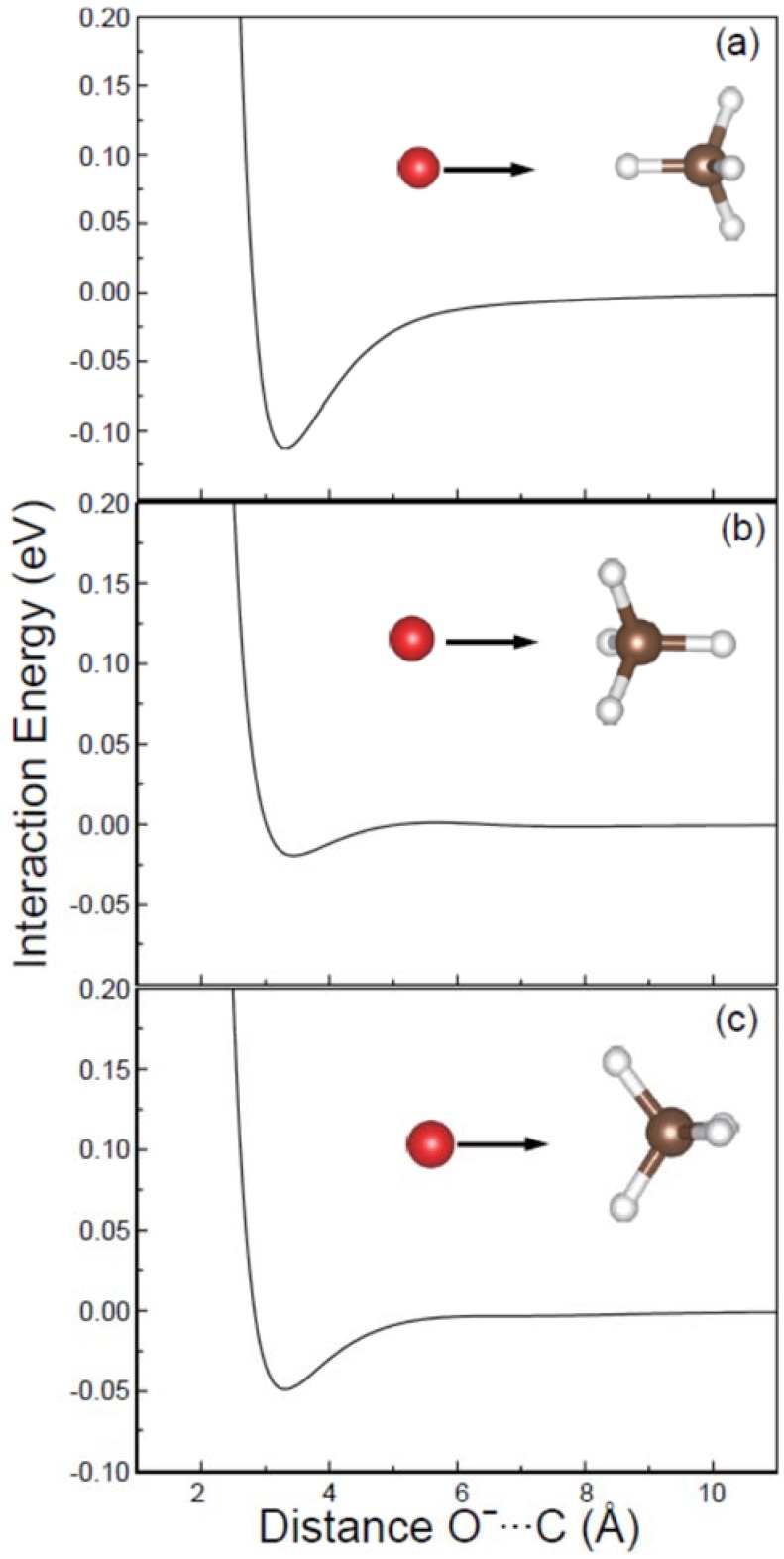
Interaction energy profiles for the attack of O^−^ (in red) to CH_4_ (H in white, C in brown) in the apex (**a**), center-of-plane (**b**) and center-of-edge-angle (**c**) directions.

As for CH_4_, it is a tetrahedron molecule characterized by T_d_ point group with four C_3_ and three C_2_ symmetrical axes. Therefore, three O^−^ attack modes can take place for the reaction between O^−^ and CH_4_. In details, as shown in Figure 1, (1) the O^−^ attacks CH_4_ along an arbitrary C_3_ axis keeping O···H-C in a straight line (abbreviated as apex attack mode); (2) the O^−^ attacks CH_4_ along an arbitrary C_3_ axis in an opposite side keeping O···C-H in a straight line (center-of-plane attack mode); (3) the O^−^ attacks CH_4_ along a C_2_ axis of H-C-H angle bisector (center-of-edge-angle attack mode). As the doorways of the reaction, these three typical O^−^ attack directions may lead to the OH^−^ product with the different momentum distributions. Moreover, besides reaction (2) which was believed as a single channel reaction between O^−^ and CH_4_ [19,20,21,22,23], it is still unknown about the existence of other possible reaction channels. To fully understand the reaction mechanism in detail, information concerning the possible reaction pathways for the reaction is indispensable. Therefore, in this study, the stereodynamics properties of the collision reaction between O^−^ and CH_4_ have been systematically investigated employing ab initio molecular dynamics simulations with the aim to provide new insights into the reaction mechanism of the title reaction.

## 2. Computational Method

Firstly, the thermochemistry calculations were carried out for reaction (2) at the second-order Møller-Plesset (MP2) [26,27,28,29,30] perturbation method with 6 − 31 + G(d) basis set. The energetic release Δ*E* of −0.23 eV is in good agreement with the previous values [19,20,21,23,24], suggesting that the MP2/6 − 31 + G(d) level is reliable in elaborating the reaction process. Excluding the influence of any electrostatic interactions between O^−^ and CH_4_ at the initial step in the dynamic simulations, we performed the interaction energy calculations for the three attack modes at the MP2/6 − 31 + G(d) level. Then, the atomic O^−^···C distance about 6 Å was used for the initial step in the simulations because the interaction between O^−^ and CH_4_ was very weak or ignorable at this distance. In these calculations, the geometry of the CH_4_ target was fixed. On the other hand, two different transition-state structures were located, and their linkages with the reactants and products were verified by the intrinsic reaction coordinate calculations [31,32].

All on-the-fly trajectory calculations for the above three O^−^ attack modes were performed by using the Born-Oppenheimer molecular dynamics (BOMD) [33,34] method at the MP2/6 − 31 + G(d) level. Two schemes have been considered in the calculations. In the first scheme, each dynamic evolution was initiated with the atomic O^−^···C distance about 6 Å between the O^−^ ion with a certain velocity or kinetic energy and CH_4_ molecule. There are no internal motions for CH_4_ at the beginning. Then, the dynamic evolution for the three attack directions (apex, center-of-plane, and center-of-edge-angle) of the incident O^−^ ion was simulated. In the other scheme, we considered the internal motions of CH_4_ that were mimicked with the atomic motions of CH_4_ at the vibrational ground state. The atomic motions or velocities of CH_4_ at the initial step of simulation were stochastically selected from the 400 fs BOMD simulations of the isolated CH_4_ at the vibrational ground state, where more details can be found in the Supporting Information (SI). In the above two schemes, the time scale of simulations was about 400 fs with a time step of 0.2 femtosecond (fs). Ten different collision energies of O^−^ ion were selected in a range of 0.5–10.0 eV, where the translational energy of CH_4_ was zero. No dissociation pathways were constrained in the simulations of about 500 trajectories. All calculations were performed with Gaussian 09 program [35].

To explore the charge transfer behavior and the net charges carried on the formed fragments in the reaction process, natural bond orbital (NBO) analyses have been performed on the basis of the optimized geometries. The contour maps of the relevant NBOs were analyzed and drawn with Multiwfn program [36].

## 3. Results and Discussion

### 3.1. Intermolecular Interactions and Transition States

The rigid potential energy profiles were obtained by scanning the O^−^···C distance by fixing the CH_4_ geometry, where the O^−^···CH_4_ interaction energies were calculated including the corrections of basis set superposition errors [37,38]. As shown in Figure 1, the interaction between O^−^ and CH_4_ is quite weak when the distance of O^−^···C is larger than 6 Å. Therefore, the following dynamics simulations will start with the distance of O^−^···C about 6 Å, ensuring no influences of the different strengths of the O^−^···CH_4_ interaction at the initial step. Furthermore, the minima on the reaction profiles were located around 3.4 Å. Meanwhile, as shown in Figure 1a, the strongest attraction of about 0.11 eV appears for the apex attack mode, implying the stabilization of the pre-reactive complex [O···HCH_3_]^−^.

**Figure 2:**
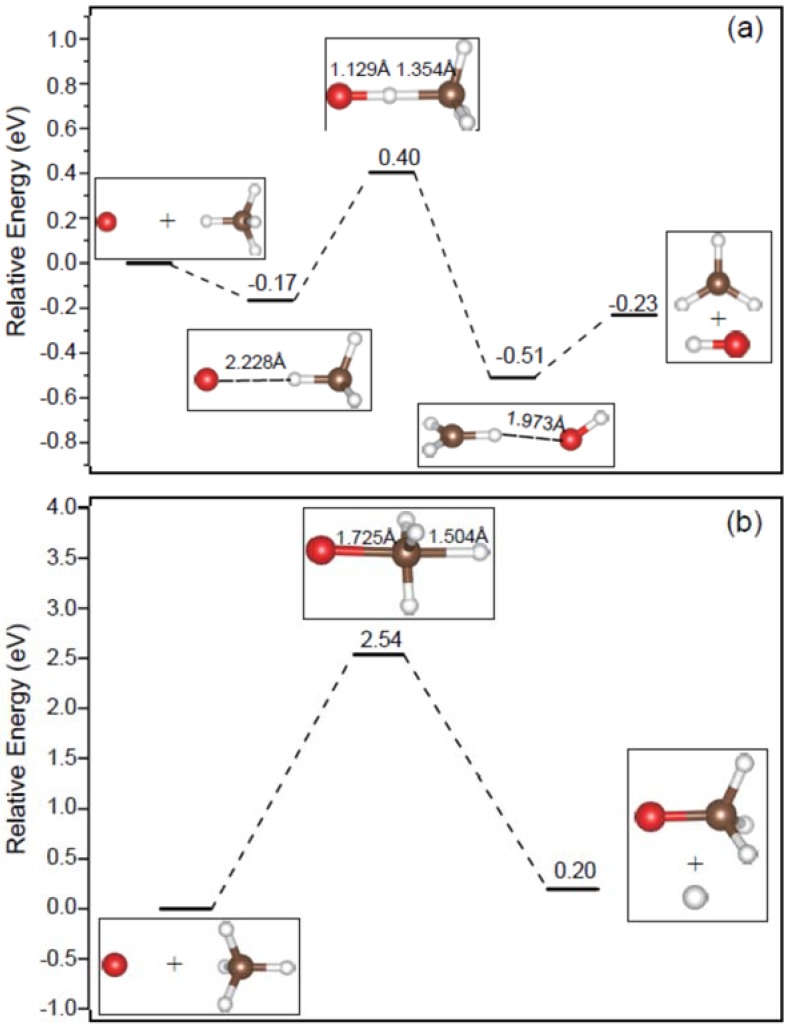
The reaction profiles for the attack of O^−^ to CH_4_ in the apex (**a**) and center-of-plane (**b**) attack modes.

For the O^−^ apex attack mode, as shown in Figure 2a, a transition state (O-H-CH_3_)^−^ possessing C_3v_ symmetry has been located after the formation of the pre-reactive complex [O···HCH_3_]^−^, which is associated with the reactants and the products of OH^−^ and CH_3_. To investigate the dependence of basis set for the transition state, larger basis sets 6 − 311 + G(3df,2p) and AUG-cc-pVTZ have been employed. As a result, the corresponding transition states possessing C_3v_ symmetry can still be obtained, where the Cartesian coordinates of them have placed in Table S1 of the SI for reference.

As for the O^−^ center-of-plane attack mode, a new reaction channel, i.e.,

O^−^ + CH_4_ → H + OCH_3_^−^ (3)

has been observed. As depicted in Figure 2b, a transition state (O-CH_3_-H)^−^ was located about 2.54 eV higher in energy than the reactants. Moreover, reaction (3) is an endothermic nucleophilic substitution (S_N_2) process (∆*E* = 0.20 eV), which has not been reported previously. In the transition state (O-CH_3_-H)^−^, the atomic distance between O and C and one C-H bond length are 1.725 and 1.504 Å, respectively.

As for the O^−^ center-of-edge-angle attack mode, no corresponding transition state has been located. 

### 3.2. Stereodynamics of the Reactions

Above quantum chemistry calculations provide us the reaction thresholds and the energy barriers on the minimum energy pathways of reactions (2) and (3). In the following BOMD simulations, the O^−^ ion with a certain kinetic energy attacks the motionless target CH_4_ from a position of 6 Å away. Note that all the energies required in the reaction including the internal energies of the products and the energy barrier to be overcome must be transformed from the kinetic energy of the incident O^−^ ion. Therefore, not all of the collisions can proceed for the O^−^ ion with the kinetic energy in the range of 0.5–10.0 eV although reaction (2) is an exothermic process. Here, three typical trajectories have been mainly discussed, where the initial kinetic energy of the incident O^−^ ion is 1.80 eV (corresponding to the initial velocity 4.66 × 10^3^ m/s), 6.60 eV (8.92 × 10^3^ m/s), and 9.20 eV (10.53 × 10^3^ m/s) for the apex, center-of-plane, and center-of-edge-angle attack modes, respectively.

**Figure 3:**
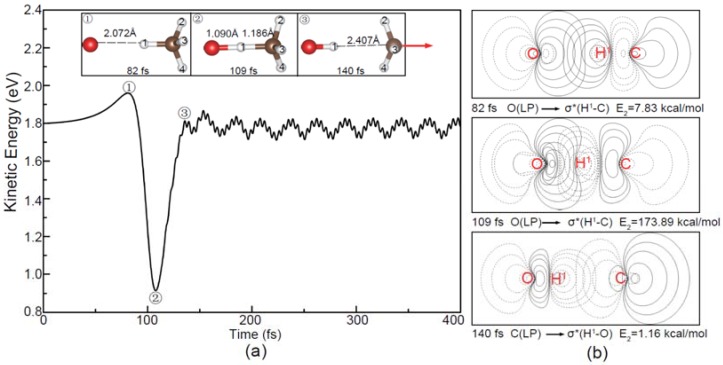
(**a**) Evolution of the total kinetic energy and snapshots in the reaction beginning with the O^−^ apex attack (1.8 eV); (**b**) The corresponding natural bond orbital (NBO) contour maps at the selected times and E_2_ is the hyperconjugative energy. The red arrow in (**a**) shows the translational direction of the product CH_3_.

As shown in Figure 3a, the O^−^ projectile is accelerated gradually in the apex direction from the initial kinetic energy 1.80 eV to 1.96 eV due to the attraction interaction depicted in Figure 1a. Then, the oxygen is combined with the hydrogen atom at 109 fs and the atomic distances of O-H^1^ and C-H^1^ reach the minima, indicating the lowest kinetic energy but the highest potential energy of the [O···HCH_3_]^−^ complex. At 140 fs, the C-H^1^ bond breaks and the CH_3_ product moves forward. After that, two products completely depart from each other. The ripples of the total kinetic energy profile in Figure 3a are attributed to the internal energy distributions of the products. As shown in Figure 3b, the NBO contour maps show the process of the negative charge transfer from O^−^. Before 109 fs, the lone pair (LP) electron of O^−^ transfers to the virtual antibonding orbital of the C-H bond followed by the formation of the O-H^1^ bond. For the sake of simplicity, the selected charge distributions of the reactants and the products in the dynamic process have been given in Table S2 of the SI for reference.

**Figure 4:**
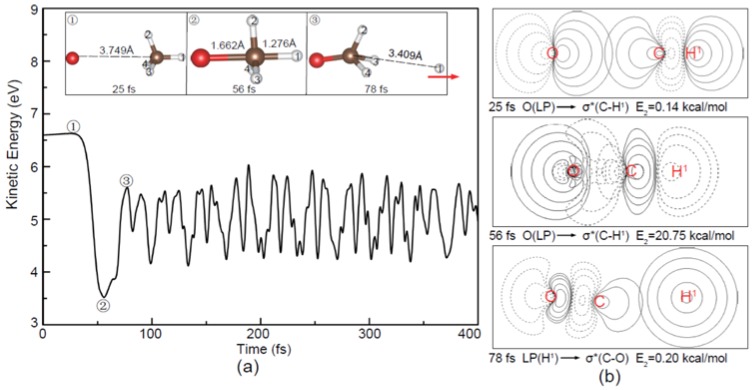
(**a**) Evolution of the total kinetic energy and snapshots in the reaction beginning with the O^−^ center-of-plane attack (6.6 eV); (**b**) The corresponding NBO contour maps at the selected times and E_2_ is the hyperconjugative energy. The red arrow in (**a**) shows the translational direction of the product H.

**Figure 5:**
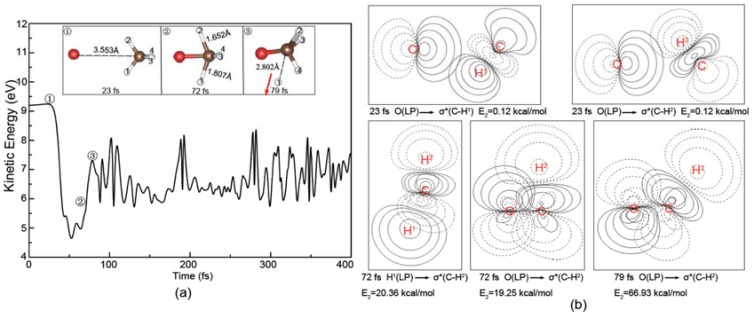
(**a**) Evolution of the total kinetic energy and snapshots in the reaction beginning with the O^−^ center-of-edge-angle attack (9.2 eV); (**b**) The corresponding NBO contour maps at the selected times and E_2_ is the hyperconjugative energy. The red arrow in (**a**) shows the translational direction of the product H.

In the center-of-plane and center-of-edge-angle attack processes, as shown in Figure 4 and Figure 5, the O^−^ is directly combined with C atom, leading to the release of H atom in the opposite direction of the O^−^ (Figure 4) and the side H atom (Figure 5), respectively. The charge transfers of LP(O^−^) → $\sigma_{C-H^{1}}^{*}$ can also be observed in these two cases. Compared with the above apex attack mode in Figure 3, remarkable variations of the total kinetic energy can be observed here, suggesting that the internal energy distributions of the products are different depending on the attack modes.

**Figure 6:**
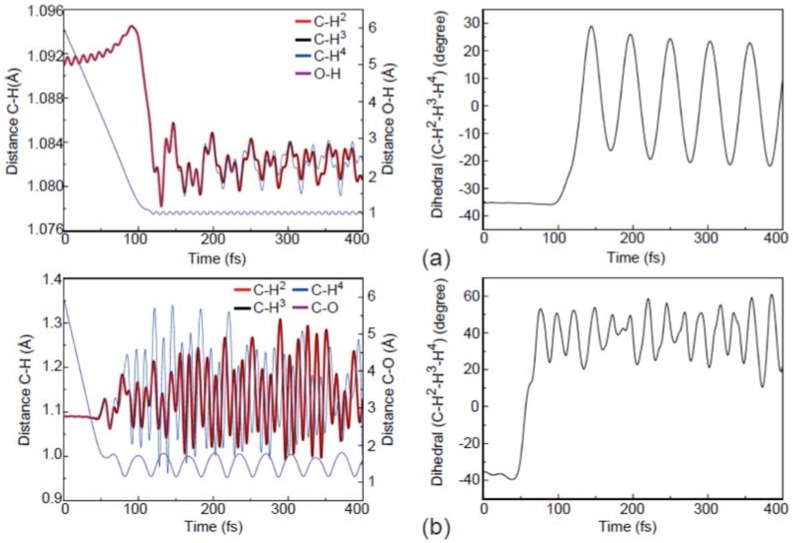

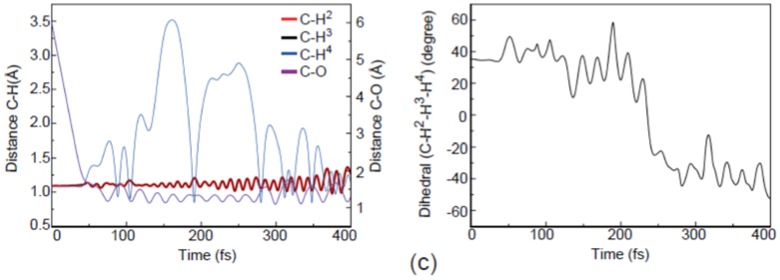
Evolution of the atomic distances and dihedral angles for the apex (**a**), center-of-plane (**b**), and center-of-edge-angle (**c**) attack modes.

As shown in Figure 6a, the apex attack reaction results in the activation of the internal motions mostly for the umbrella vibrational mode of the methyl radical. On the other hand, as shown in Figure 6b,c, the internal motions of OCH_3_^−^ are distinctly different. In particular, the stretching motion of the C-H, the umbrella motion of CH_3_, and the stretching motion of OH^−^ are much more active in the reaction induced by the center-of-plane attack. As a result, as can be seen from the stereodynamics effects for the above three typical trajectories, the sites of the H atom released and the internal energy distributions of the products are highly dependent on the O^−^ attack directions. Here, it should be noted that the present simulations are performed for the collisions along the different attractive interaction lines (i.e., the impact parameter *b* is zero). Meanwhile, the geometry of the target CH_4_ is motionless initially in the simulations.

As shown in Figure 2a, reaction (2) is a double-well reaction. In comparison with the mechanisms proposed for the double-well S_N_2 anion–molecule reactions [39], the apex and center-of-plane attack modes correspond to the reactions involving the formation of the frontside complex. While the center-of-edge-angle attack mode is similar to those of the S_N_2 reactions leading to the formation of the ion-dipole complex. Certainly, there are other mechanisms for the S_N_2 reactions [39,40], largely depending on the impact parameter and the internal atomic motions of the target molecule.

### 3.3. Reactions with Thermally Vibrating CH_4_

As mentioned above, the internal motion (molecular vibration) and the rotation of the target molecule can influence the collision process [11,19,20,21]. To further confirm this point, the reaction of O^−^ with thermally vibrating CH_4_ has also been investigated. As shown in Figure S1 of the SI, we stochastically select 50 geometries of CH_4_ from thermally equilibrated simulation after 200 fs at an interval of 4 fs. At the initial steps of the reaction simulations, the atomic coordinates and velocities of oxygen and carbon atom are set up in the center-of-mass Cartesian coordinate (Tables S3 and S4 of the SI), where the O^−^ kinetic energies are 0.5 (2.46 × 10^3^ m/s), 1.0 (3.47 × 10^3^ m/s), 1.5 (4.25 × 10^3^ m/s), 2.0 (4.91 × 10^3^ m/s), 2.5 (5.49 × 10^3^ m/s), 3.0 (6.01 × 10^3^ m/s), and 3.5 eV (6.50 × 10^3^ m/s), respectively.

**Figure 7:**
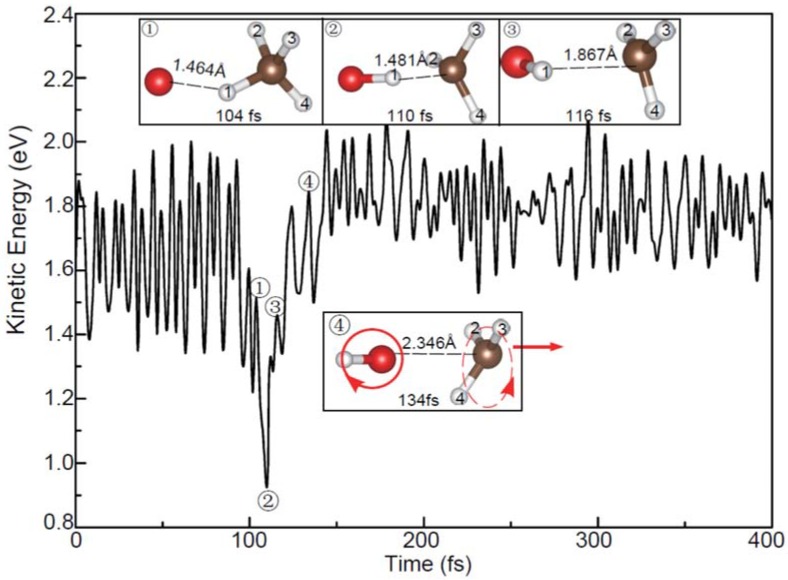
Evolution of the total kinetic energy and snapshots in the reaction of the vibrating CH_4_ with the O^−^ with the kinetic energy of 1.0 eV. The circle and the arrow represent the molecular rotating and moving directions.

**Figure 8:**
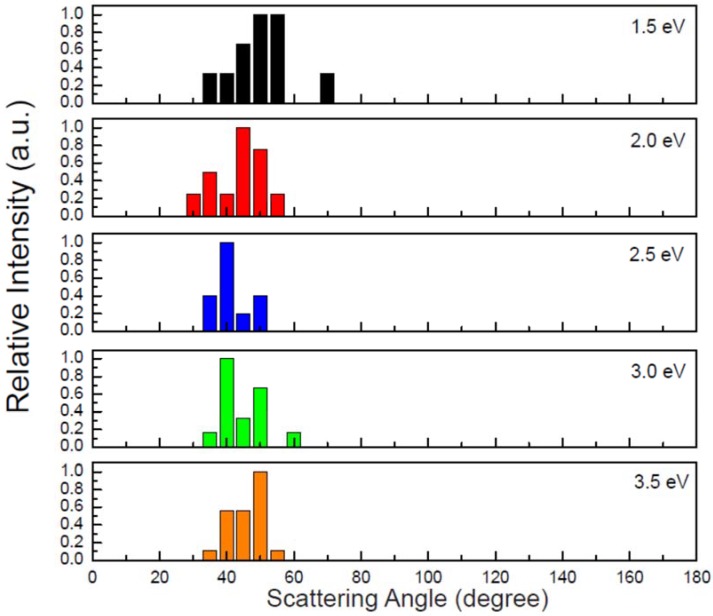
Angular distributions of the OH^−^ product. The relative intensities are normalized with the respective maximum values.

Similar to the above reaction, reaction (2) is also the predominant pathway here. As shown in Figure 7, the reaction of the O^−^ having the collision energy of 1.0 eV leads to OH^−^ and CH_3_ products via a direct H-abstraction. However, the detailed process is distinctly different from that observed in Figure 3a although the charge transfers depicted in Figure 3b and Figure S2 of the SI are similar to each other. As shown in Figure S3 of the SI, the C-H^2,3,4^ bond stretching motions are more significant. Moreover, the molecular rotations for both OH^−^ and CH_3_ are remarkable. In view of the fact that the excess energy of the reaction can be redistributed to the internal atomic motions and molecular rotations, the translational energies of the products at this collision energy (1.0 eV) are relatively small. At the higher collision energy, the products can obtain more translational energies and the angular distributions of OH^−^ are anisotropic. The calculated relative intensities of the scattering angular distributions (in laboratory coordinate) of OH^−^ have been plotted in Figure 8. Obviously, the maxima are distributed between 40° and 60°, which is generally in agreement with experimental results [23].

**Figure 9:**
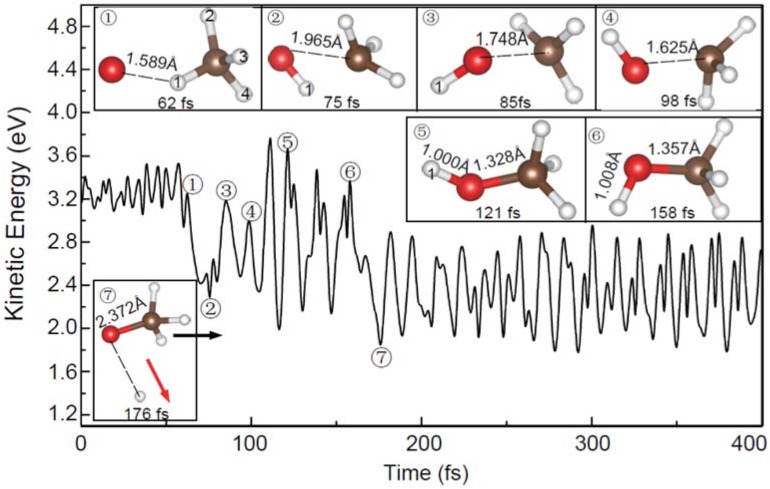
Evolution of the total kinetic energy and snapshots in the roaming reaction with the vibrating CH_4_ at the O^−^ kinetic energy of 2.5 eV. The arrows represent the molecular moving directions.

In addition, a few new trajectories leading to the products of OCH_3_^−^ and H have also been observed. As shown in Figure 9, the dynamic process of reaction (3) is completely different from those observed in Figure 4a and Figure 5a. In this process, an H atom is firstly abstracted to form the slowly rotating OH^−^. Then, this OH^−^ group is recombined with CH_3_. Finally, the H atom attached to the O atom is released and the vibrating OCH_3_^−^ is formed. Therefore, the above phenomena suggest that the reaction (3) is a typical roaming process, which has also been observed in the molecular dynamics of the other systems [39,40,41,42]. Given the fact that no roaming mechanism for reactions (3) has been reported previously, further experimental and theoretical studies are required to confirm this point.

## 4. Conclusions

In this study, the reaction between O^−^ and CH_4_ has been systematically investigated using the ab initio molecular dynamics simulations. The stereodynamics properties for the different attack modes of O^−^ to the initially motionless CH_4_ have been revealed. As a result, besides the major exothermic pathway to produce the OH^−^ and CH_3_, a new endothermic channel into the yields of H and OCH_3_^−^ has been observed. Meanwhile, the translational and internal energies of the products are dependent on the attack modes of O^−^. Moreover, considering the internal atomic motions of the gas-phase target CH_4_, we also performed the dynamics simulations for the reaction between O^−^ and the thermally vibrating CH_4_. It was shown that most of the trajectories can lead to the production of OH^−^ and CH_3_, and the radical products are populated at the ro-vibrational states and in the forward distributions. Especially, it is worth noting that a typical roaming pathway to produce the H and OCH_3_^−^ has also been observed, which deserves further explorations in the future.

## Supplementary Materials

The following are available online, Figure S1: Variation of the kinetic energy for the equilibration process of the isolated CH_4_ at the vibrational ground state, Figure S2: NBO contour maps of the charge transfers in one of the trajectory of the O^−^ (with the kinetic energy of 1.0 eV) reaction with the vibrating CH_4_. E_2_ is the hyperconjugative energy and represents the strength of orbital-orbital interaction, Figure S3: Evolution of the C-H atomic distance and dihedral angle in one of the trajectory of the O^−^ (with the kinetic energy of 1.0 eV) reaction with the vibrating CH_4_, Figure S4: Atomic labels of methane molecule used in Tables S2 and S3, Table S1: The Cartesian coordinates of the transition state possessing C3v symmetry in the major pathway of the title reaction, Table S2: Charge distributions of the reactants and the products in the reactions of O^−^ with the initially fixed-structure CH_4_. The charge values are obtained with natural bond orbital analysis, Table S3: Geometric parameters of methane molecule at the 10 moments of vibration, Table S4: The speeds (m/s) of individual atoms of methane molecule at the 10 moments of vibration.
