# Supplementary material for: Ab Initio Molecular Dynamics Simulation Study on the Stereo Reactions between Atomic Oxygen Anion and Methane
Source: Molecules. 2018 Sep 29;23(10):2495. doi: 10.3390/molecules23102495 (PMC6222880; doi:10.3390/molecules23102495)
Supplement: Supplementary file 1 [file molecules-23-02495-s001.pdf]

Supporting Information for

Molecular Dynamics Simulation Study of the Stereo Reactions between Atomic Oxygen Anion and Methane

Weihua Wang, Wenling Feng*, Wenliang Wang, Ping Li*

Key Laboratory of Life-Organic Analysis, School of Chemistry and Chemical Engineering,

Qufu Normal University, Qufu 273165, People's Republic of China

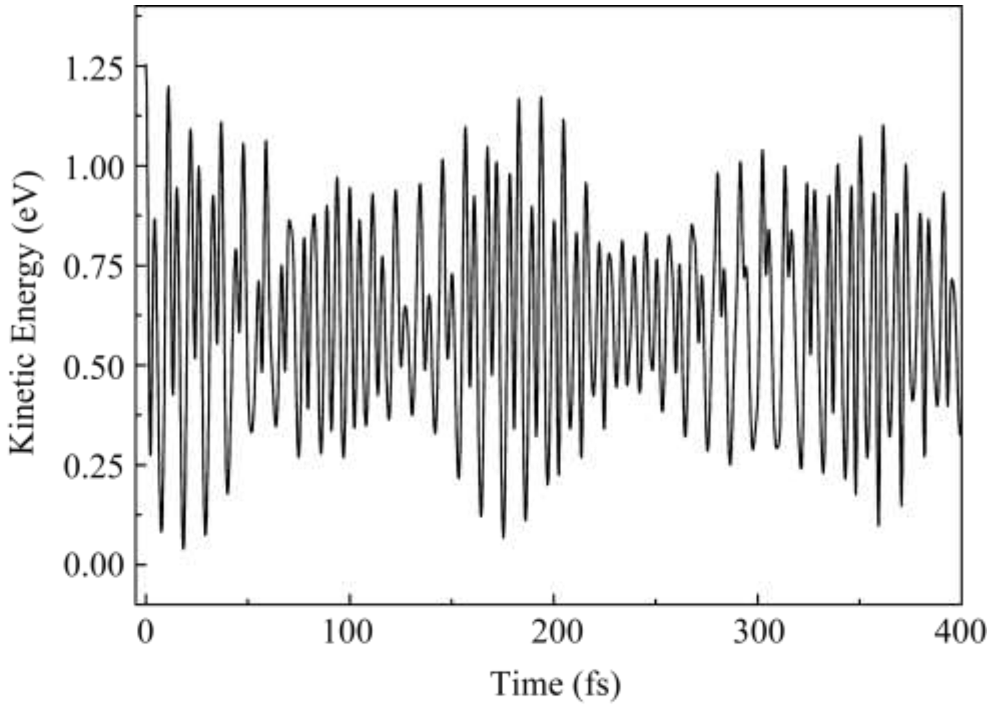

Figure S1. Variation of the kinetic energy for the equilibration process of the isolated CH_4 at the vibrational ground state.

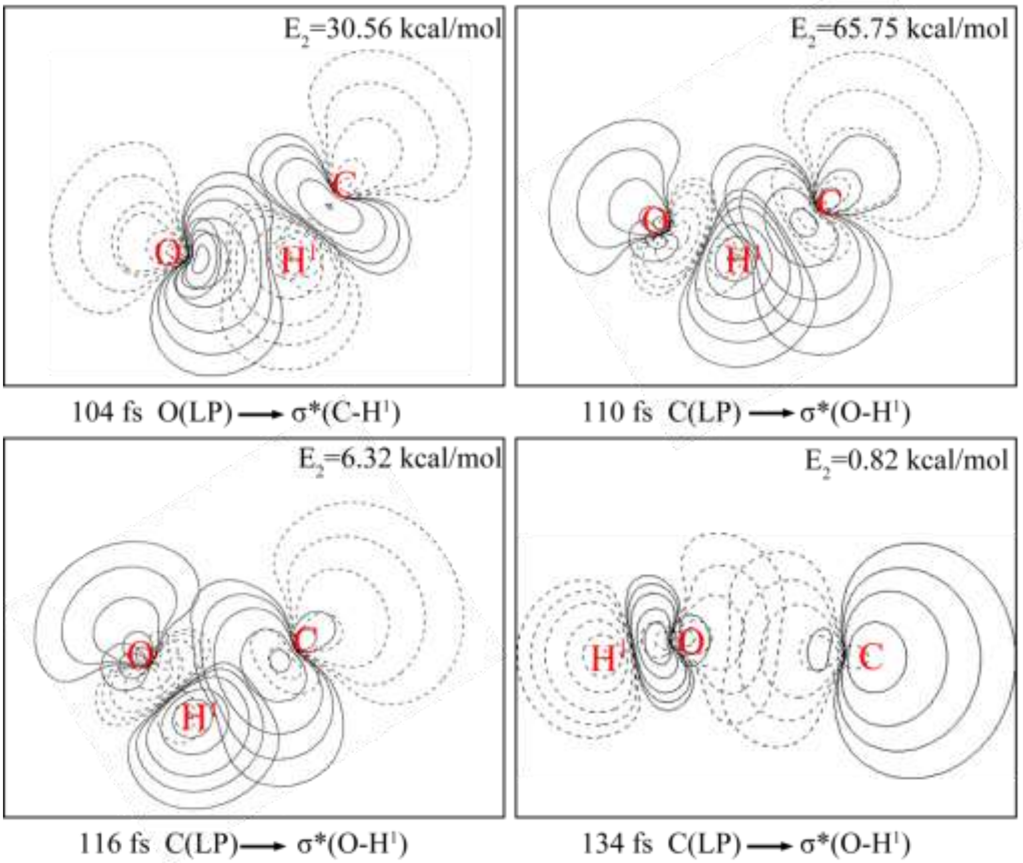

Figure S2. NBO contour maps of the charge transfers in one of the trajectory of the O^- (with the kinetic energy of 1.0 eV) reaction with the vibrating CH_4. E_2 is the hyperconjugative energy and represents the strength of orbital-orbital interaction.

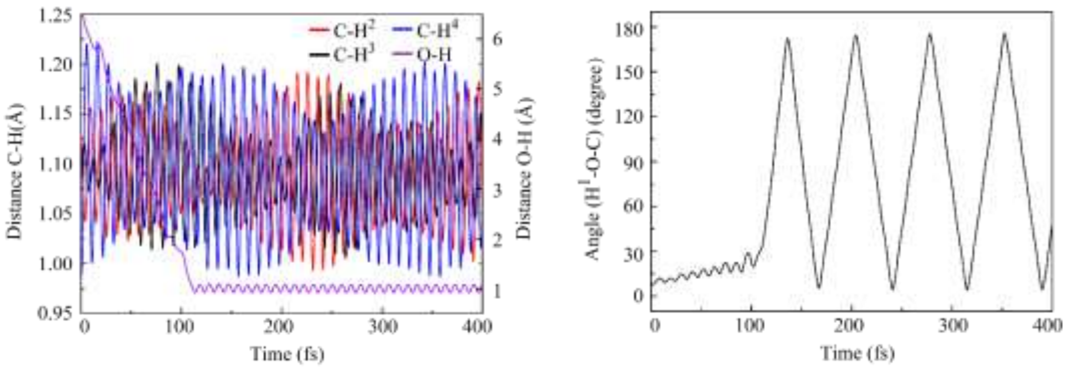

Figure S3. Evolution of the C-H atomic distance and dihedral angle in one of the trajectory of the O$^-$ (with the kinetic energy of 1.0 eV) reaction with the vibrating CH_4.

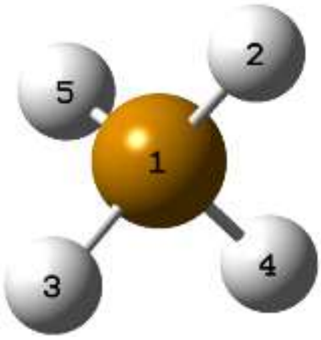

Figure S4. Atomic labels of methane molecule used in Tables S2 and S3.

Table S1. The Cartesian coordinates of the transition state possessing C_{3v} symmetry in the major pathway of the title reaction

MP2/6-311+G(3df,2p)				MP2/AUG-cc-pVTZ			
6	0.000000	0.000000	−1.110721	6	0.000000	0.000000	−1.112574
1	0.000000	1.041154	−1.419336	1	0.000000	1.042241	−1.418585
1	0.901666	−0.520577	−1.419336	1	0.902607	−0.521120	−1.418585
1	−0.901666	−0.520577	−1.419336	1	−0.902607	−0.521120	−1.418585
1	0.000000	0.000000	0.196051	1	0.000000	0.000000	0.194466
8	0.000000	0.000000	1.340785	8	0.000000	0.000000	1.342091

Table S2. Charge distributions of the reactants and the products in the reactions of O^{-} with the initially fixed-structure CH_4. The charge values are obtained with natural bond orbital analysis

apex attack	fragment charge		center-of-plane attack	fragment charge		center-of-edge-angle attack	fragment charge	
fs	O	CH_4	fs	O	CH_4	fs	O	CH_4
0	-1.000	0.000	0	-0.999	-0.001	0	-1.000	-0.000
82	-0.968	-0.032	25	-0.998	-0.002	23	-0.996	-0.004
109	-1.016	0.016	56	-0.939	-0.061	64	-1.037	0.037
	OH	CH_3		OCH_3	H		OCH_3	H
140	-0.994	-0.006	78	-1.000	0.000	79	-0.992	-0.008

Table S3. Geometric parameters of methane molecule at the 10 moments of vibration*

CH_4	bond distance (Å)				dihedral angle (˚)			
	C^1-H^2	C^1-H^3	C^1-H^4	C^1-H^5	C^1-H^2-H^3-H^4	C^1-H^2-H^3-H^5	C^1-H^2-H^4-H^5	C^1-H^3-H^4-H^5
200 fs	1.089	1.067	1.132	1.163	32.4	-40.1	41.0	-24.0
220 fs	1.183	1.151	1.274	1.181	35.2	-37.5	37.2	-31.0
240 fs	1.178	1.136	1.243	1.097	39.1	-32.8	32.6	-39.7
260 fs	1.130	1.069	1.095	0.948	40.9	-30.5	30.1	-40.4
280 fs	1.104	1.078	0.996	1.636	37.2	-38.0	34.6	-33.6
300 fs	1.129	1.105	0.996	1.239	33.8	-46.5	39.6	-22.2
320 fs	1.142	1.123	1.088	1.307	33.1	-48.4	41.3	-22.2
340 fs	1.097	1.118	1.151	1.212	34.8	-42.8	38.6	-32.5
360 fs	1.037	1.073	1.149	0.954	39.4	-32.9	32.1	-36.9
380 fs	1.035	1.090	1.192	1.044	41.1	-31.4	33.9	-36.5

*Atomic labels see Figure S4.

Table S4. The speeds (m/s) of individual atoms of methane molecule at the 10 moments of vibration*

200 fs	X(*10^3)	Y(*10^3)	Z(*10^3)	220 fs	X(*10^3)	Y(*10^3)	Z(*10^3)
C^1	0.600	-0.168	0.321	C^1	0.189	-0.628	0.074
H^2	-4.581	-5.198	-3.559	H^2	-3.594	-2.812	-1.582
H^3	3.880	2.639	-1.538	H^3	2.345	3.228	2.548
H^4	-6.655	3.365	3.303	H^4	-0.284	2.381	0.578
H^5	0.203	1.198	-2.029	H^5	-0.717	4.685	-2.421
240 fs	X(*10^3)	Y(*10^3)	Z(*10^3)	260 fs	X(*10^3)	Y(*10^3)	Z(*10^3)
C^1	0.106	-0.328	0.333	C^1	-0.254	0.581	0.445
H^2	-1.149	-0.092	1.367	H^2	0.968	0.676	2.686
H^3	-0.905	0.096	4.228	H^3	-0.655	-0.572	0.066
H^4	4.516	-1.778	-3.819	H^4	3.064	-4.591	-5.858
H^5	-3.721	5.678	-5.741	H^5	-0.351	-2.436	-2.198
280 fs	X(*10^3)	Y(*10^3)	Z(*10^3)	300 fs	X(*10^3)	Y(*10^3)	Z(*10^3)
C^1	-0.394	1.103	0.244	C^1	0.081	0.422	-0.022
H^2	1.672	-0.243	1.842	H^2	1.812	-0.365	0.754
H^3	0.542	-0.105	-3.798	H^3	0.052	-0.031	-2.652
H^4	-1.645	-2.833	-3.381	H^4	-4.631	1.437	1.097
H^5	4.121	-9.962	2.425	H^5	1.798	-6.063	1.066
320 fs	X(*10^3)	Y(*10^3)	Z(*10^3)	340 fs	X(*10^3)	Y(*10^3)	Z(*10^3)
C^1	0.238	-0.720	-0.432	C^1	0.326	-1.330	-0.376
H^2	1.843	1.301	0.603	H^2	1.989	2.965	1.026
H^3	-0.559	1.274	0.413	H^3	-2.076	0.815	3.025
H^4	-3.102	4.516	3.845	H^4	1.321	3.904	3.036
H^5	-1.018	1.489	0.279	H^5	-5.122	8.153	-2.604
360 fs	X(*10^3)	Y(*10^3)	Z(*10^3)	380 fs	X(*10^3)	Y(*10^3)	Z(*10^3)
C^1	0.307	-0.723	-0.123	C^1	-0.215	0.623	-0.309
H^2	1.249	1.966	0.062	H^2	0.343	-0.206	-1.500
H^3	-2.049	0.052	0.808	H^3	-0.255	0.086	-3.879
H^4	1.058	2.117	2.030	H^4	-2.148	0.306	1.632
H^5	-3.917	4.482	-1.429	H^5	4.619	-7.612	7.430

*Atomic labels see Figure S4.
